# Novel Mutations in *BMPR2, ACVRL1* and *KCNA5* Genes and Hemodynamic Parameters in Patients with Pulmonary Arterial Hypertension

**DOI:** 10.1371/journal.pone.0100261

**Published:** 2014-06-17

**Authors:** Guillermo Pousada, Adolfo Baloira, Carlos Vilariño, Jose Manuel Cifrian, Diana Valverde

**Affiliations:** 1 Department of Biochemistry, Genetics and Immunology, Faculty of Biology, University of Vigo, Instituto de Investigación Biomédica de Vigo (IBIV), Vigo, Spain; 2 Respiratory Division, Complejo Hospitalario Universitario de Pontevedra, Pontevedra, Spain; 3 Respiratory Division, Complejo Hospitalario Universitario de Vigo, Vigo, Spain; 4 Respiratory Division, Hospital Universitario Marqués de Valdecilla, Santander, Spain; University of Colorado, Denver, United States of America

## Abstract

**Background:**

Pulmonary arterial hypertension (PAH) is a rare and progressive vascular disorder characterized by increased pulmonary vascular resistance and right heart failure. The aim of this study was to analyze the Bone Morphogenetic Protein Receptor 2 (*BMPR2*), Activin A type II receptor like kinase 1 (*ALK1/ACVRL1*) and potassium voltage-gated channel, shakerrelated subfamily, member 5 (*KCNA5*) genes in patients with idiopathic and associated PAH. Correlation among pathogenic mutations and clinical and functional parameters was further analyzed.

**Methods and Results:**

Forty one patients and fifty controls were included in this study. Analysis of *BMPR2, ACVRL1* and *KCNA5* genes was performed by polymerase chain reaction (PCR) and direct sequencing. Fifty one nucleotide changes were detected in these genes in 40 of the 41 patients; only 22 of these changes, which were classified as pathogenic, have been detected in 21 patients (51.2%). Ten patients (62.5%) with idiopathic PAH and 10 (40%) with associated PAH showed pathogenic mutations in some of the three genes. Several clinical and hemodynamics parameters showed significant differences between carriers and non-carriers of mutations, being more severe in carriers: mean pulmonary artery pressure (p = 0.043), pulmonary vascular resistence (p = 0.043), cardiac index (p = 0.04) and 6 minute walking test (p = 0.02). This differences remained unchanged after adjusting for PAH type (idiopathic vs non idiopathic).

**Conclusions:**

Pathogenic mutations in *BMPR2* gene are frequent in patients with idiopathic and associated PAH group I. Mutations in *ACVRL1* and *KCNA5* are less frequent. The presence of these mutations seems to increase the severity of the disease.

## Introduction

Pulmonary arterial hypertension (PAH; OMIM #178600) is a rare and progressive disorder characterized by obstruction of pre-capillary pulmonary arteries [1]. It is defined by a sustained increase in mean pulmonary artery pressure (mPaP) ≥25 mmHg at rest with normal wedge pressure [2]. Symptoms of PAH include dyspnea, syncope and chest pain, and eventually leads to right-sided heart failure and death [1]. Structural and functional changes in the vascular wall and thrombus formation are the main factors responsible for the increased pulmonary vascular resistance in these patients [3].

PAH can be inherited (FPAH), idiopathic (IPAH), or associated with other diseases, drug or toxin exposures (APAH) [4]. The disease is more frequent in women, with a ratio of at least 1.7∶1 women to men [2]. Much of what is known about the genetic basis of PAH is related to mutations in bone morphogenetic protein receptor type 2 (*BMPR2*). This gene is located on chromosome 2q33 and mutations have been identified in over 80% of patients with FPAH, but are likely to be responsible for over 90% of the cases. However, only 20% of carriers developed the disease [5], [6]. The frequency of mutations in *BMPR2* gene is not well defined in IPAH, but it has been reported a value of 9–26% in small cohorts of patients [6], [7], [8].

Some other genes have been implicated in the pathogenesis of the disease, including Activin A type II receptor like kinase 1 (*ALK1/ACVRL1*) and potassium voltage-gated channel, shakerrelated subfamily, member 5 (*KCNA5*). Mutations in *ACVRL1* gene, located in chromosome 12q13, are directly related to some cases of PAH associated with hereditary hemorrhagic telangiectasia (HHT) [9]. This receptor is also a member of the transforming growth factor beta (TGF-β) superfamily and plays a role in different tissues producing different responses, including proliferation, differentiation, migration, increase of cell survival and angiogenesis. *ACVRL1* is expressed mainly in the developing vascular system and plays a critical role in arteriogenesis and developing arterial endothelial cells [10], [11]. *KCNA5* protein is situated in the cellular intermembrane space and is composed by four subunits. The *KCNA5* gene is located on chromosome 12p13 and is formed by a single exon of 2865 bp and 613 residues. Indeed, mutations in the exon or in the promoter region of *KCNA5* gene have been reported to be associated with IPAH and may underline the altered function and expression of voltage-gated K+ channel 1.5 (Kv1.5) observed in pulmonary arteriolar smooth muscle cells (PASMC) from these patients [12], [13].

The aim of this study was to analyze *BMPR2, ACVRL1* and *KCNA5* genes in patients with idiopathic and associated PAH, characterize the changes found and correlate them with clinical and hemodynamic variables.

## Materials and Methods

### Patients and samples

Patients with idiopathic or associated PAH (group 1 of Dana Point) followed in our clinic of PAH were included in this study. Cardiac catheterization was performed using the latest consensus diagnostic criteria of the ERS-ESC (European Respiratory Society-European Society of Cardiology) (mean resting pulmonary pressure ≥25 mmHg, capillary pressure <15 mmHg) in all cases, [14]. PAH was considered idiopathic after exclusion of any of the possible causes associated with the disease. Clinical history included use of drugs, especially appetite suppressants, and screening for connective tissue diseases and hepatic disease. The study included serology for HIV, autoimmunity, thoracic CT scan and echocardiography. Patients with PAH that could be related to chronic lung disease were excluded. Fifty healthy individuals were used as controls. All patients and controls signed an informed consent. The Autonomic Ethics Committee approved the study (*Comité Autonómico de Ética da Investigación de Galicia - CAEI de Galicia*).

Genomic DNA was extracted from leukocytes isolated from venous blood using the FlexiGene DNA Kit (Qiagen, Germany) according to the manufacturer’s protocol.

### Genomic study

Amplification of the exons and intronic junctions of the genes was performed with 50 ng of genomic DNA from each individual. Changes in other regions were not analyzed. The primers used for the *BMPR2* gene were as described by Deng *et al* [15]. The amplification conditions were as follows: 95°C for 5 min, 35 cycles of 95°C for 30 s, 55°C for 30 s (for the exons 1, 3, 5, 6, 7, 8, 9, 10, 11 and 13) and 60°C for 30 s (for the exons 2 and 12), 72°C for 30 s and, finally, 72°C for 7 min. The PCR mix contained 1.5 mM Cl_2_ Mg, with 0.2 U of Taq Polymerase (Biotaq, Bioline, UK).

The exons and intronic junctions of the *ACVRL1* gene were amplified using the conditions of 95°C for 1 min followed by 35 cycles of 95°C for 30 s, 55°C for 30 s (for the exons 5, 7, 8, 9 and 10), 56°C for 30 s (for the exon 4), 60°C for 30 s (for the exon 6), 62°C for 30 s (for the exon 2) and 64°C for the exon 3), 72°C for 30 s and, finally, 72°C for 7 min. The primers used to amplify this gene were described by Berg *et al* [16].

The single exon and intronic junctions of the *KCNA5* gene was amplified for the 4 validated amplicons spanning the coding sequence of *KCNA5* gene. The primers used were described by Tao Yang *et al* [17]. The amplification conditions were 95°C for 30 s, followed by 35 cycles of 95°C for 30 s, 66°C for 30 s, 72°C for 30 s and, finally, 72°C for 7 min.

PCR products were confirmed by electrophoresis on a 2% agarose gel containing ethidium bromide. PCR fragments were purified using the Nucleic Acid and Protein Purification kit (NucleoSpin Extract II; Macherey-Nagel, Germany) and sequenced with the BigDye Terminator version 1.1 Cycle Sequencing Kit (Applied Biosystems, California, USA). The reactions were performed on a GeneAmp PCR System 2700 (Applied Biosystems). The sequencing reactions were precipitated and finally analyzed on an ABI PRISM 3100 genetic analyzer (Applied Biosystems).

Sequence data was aligned to reference Ensembl cDNA sequence ENST00000374580 for *BMPR2* gene, ENST00000388922 for *ACVRL1* gene and ENST00000252321 for the *KCNA5* gene and examined for sequence variations. Detected mutations were confirmed by a second independent PCR reaction and were identified in both forward and reverse strands. To predict whether a rare missense variant was deleterious, we used the combined results of three different computer algorithms:

The polymorphism phenotyping program, PolyPhen-2 (available at http://genetics.bwh.harvard.edu/pph/) uses sequence conservation, structure and SWISS-PROT annotation to characterize an amino acid substitution as benign, possibly damaging or probably damaging [18].
Pmut (available at http://mmb2.pcb.ub.es:8080/PMut/) provides prediction by neural networks, which use internal databases, secondary structure prediction and sequence conservation. This program provides a binary prediction of “neutral” or “pathologic” [19].
Sort Intolerant from Tolerant (SIFT) (available at http://sift.jcvi.org) uses sequence homology to predict whether a change is tolerated or damaging [20].

Intronic, isocoding and missense changes were analyzed using the programs *NNSplice* (http://fruitfly.org:9005/seq_tools/splice.html), *NetGene2* (http://www.cbs.dtu.dk/services/NetGene2/), *Splice View* (http://zeus2.itb.cnr.it/~webgene/wwwspliceview_ex.html) and *HSF Human* (http://www.umd.be/HSF/) in order to predict whether those changes could be affecting, creating or eliminating donor/acceptor splice sites.

### Statistical analysis

Values are expressed as mean±SD (standard deviation). A non-parametric test was used for comparisons between patients and controls. Chi-square test was used to compare genotype with clinical and hemodynamic variables. These correlations were analyzed by the Spearman test. Values <0.05 were considered statistically significant. Analysis was carried out using the statistical package SPSS v19.

## Results

### Description of the cohort

Forty one unrelated PAH patients (16 idiopathic, 17 associated to connective tissue disease, 4 related to HIV and 4 porto-pulmonary) and fifty healthy controls without familial history of PAH were included. At the time of diagnosis 3 patients were in functional class (FC) I, 14 patients in FC II, 21 patients in FC III and 3 in FC IV. The clinical features of patients are showed in Table 1.

**Table 1:** Clinical features and hemodynamic parameters of patients.

	Carriers of mutations	Non-carriers of mutations	*p-value*	*Idiopathic PAH*	*Associated PAH*	*p-value*
***Number***	20	21		16	25	
***Gender***	10 F/10 M	10 F/11 M	0.216	8 F/8 M	13 F/12 M	0.552
***Age at diagnosis (years)***	53±15	51±16	0.437	52±21	53±12	0.552
***mPaP (mmHg)***	57±15	45±14	0.043	52±16	47±13	0.510
***sPaP (mmHg)***	69±22	73±19	0.448	74±20	70±21	0.490
***PVR (mmHg.l*** **^−^** ^***1***^ ***.m*** **^−^** ^***1***^ ***)***	11.92±3.18	8.53±4.46	0.043	9.96±4.68	7.21±2.8	0.222
***CI (l.m*** **^−^** ^***1***^ ***.m*** **^−^** ^***2***^ ***)***	2.05±0.68	3.75±0.44	0.040	2.6±0.74	2.54±0.45	0.346
***6MWT (m)***	314±130	428±103	0.020	370±136	374±127	0.308
***PAH types***	10 IPAH/10 APAH	6 IPAH/15 APAH	0,222	16 patients	25 patients	0,222

Values are expressed as mean ± standard deviation; F: female, M: male; mPaP: mean pulmonary artery pressure; sPaP: systolic pulmonary artery pressure; PVR: pulmonary vascular resistence; CI: cardiac index; 6MWT: 6 minute walking test; IPAH: idiopathic pulmonary arterial hypertension; APAH: associated pulmonary arterial hypertension.

### Mutations in *BMPR2*, *ACVRL1* and *KCNA5* genes

A total of 53 nucleotide changes in the *BMPR2*, *ACVRL1* and *KCNA5* genes were identified in 40 out of 41 patients. We found 30 changes in 33 patients in *BMPR2* gene, 11 variations in 24 patients in *ACVRL1* gene, and 12 changes in 15 patients in the case of *KCNA5* gene.

**Figure 1:**
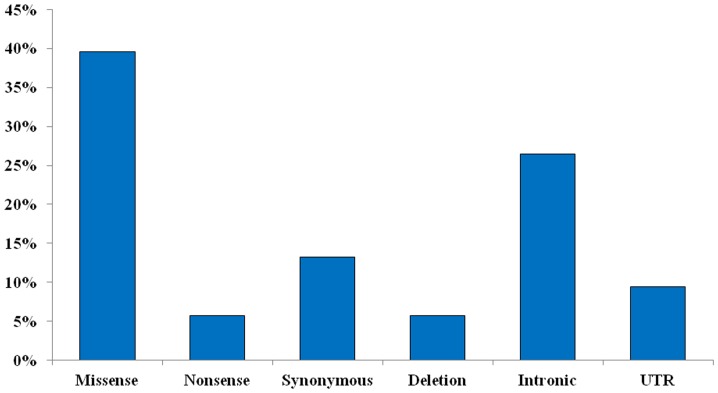
Total percentage of nucleotide changes found in this study for the analyzed genes. The variations that appear in greater proportion are missense, followed by those located in the intronic region.

Thirty-one variations (60.8%) were located in coding regions. Missense variations accounted for 39.6% of total changes found in coding regions; nonsense variations only represented 5.7% and synonymous changes a 13%. The 26.4% of changes were located in intronic regions (two of them were heterozygous deletions) and a 9.4% in UTR regions of these genes. (Figure 1).

The three different computer algorithms used to classify the nucleotide changes showed different results. A missense mutation was considered pathogenic when at least two of the three programs (*PolyPhen-2*, *Pmut* and *SIFT*) classified it as pathogenic. These changes are summarized in Table 2. None of these mutations were detected in a panel of 100 chromosomes from controls. Furthermore, we used four programs to predict whether these changes could affect donor/acceptor splice sites. We consider a mutation as potentially pathogenic when the pathogenic score was equal or greater than 2 (Tables 3, 4, 5).

**Table 2:** Missense changes found in the coding region of the *BMPR2, ACVRL1* and *KCNA5* genes and their classification according to three different computer algorithms (*PolyPhen-2*, *Pmut* and *SIFT*).

CLASSIFICATION MISSENSE VARIATIONS FOUND IN THE CODING REGION OF GENES							
*Gene*	*Nucleotide change*	*Amino-acid change*	*Times founded*	*PolyPhen-2*	*Pmut*	*Sift*	*Score*
*BMPR2 (Exon 2)*	c.190A>C	p.(S64G)	1	Benign	Neutral	Tolerated	0
*BMPR2 (Exon 2)*	c.229A>T	p.(I77L)	1	Benign	Neutral	Damaging	1
*BMPR2 (Exon 3)*	c.251G>T	p.(C84F)	2	Probably damaging	Neutral	Damaging	2
*BMPR2 (Exon 3)*	c.259C>T	p.(H87Y)	1	Benign	Neutral	Damaging	1
*BMPR2 (Exon 3)*	c.275A>T	p.(Q92L)	1	Benign	Pathologic	Damaging	2
*BMPR2 (Exon 4)*	c.484G>C	p.(A162P)	1	Probably damaging	Neutral	Damaging	2
*BMPR2 (Exon 6)*	c.790G>A	p.(D264N)	1	Possibly damaging	Neutral	Damaging	2
*BMPR2 (Exon 8)*	c.1021G>A	p.(V341M)	3	Possibly damaging	Neutral	Damaging	2
*BMPR2 (Exon 12)*	c.2324G>A	p.(S775N)	2	Benign	Neutral	Tolerated	0
*ACVRL1 (Exon 2)*	c.24A>T	P(.K8N)	1	Benign	Neutral	Tolerated	0
*ACVRL1 (Exon 3)*	c.176A>T	p.(E59V)	1	Benign	Neutral	Tolerated	0
*ACVRL1 (Exon 4)*	c.476A>T	p.(E159V)	1	Benign	Neutral	Tolerated	0
*ACVRL1 (Exon 6)*	c.673A>T	p.(S225C)	1	Probably Damaging	Pathological	Damaging	3
*ACVRL1 (Exon 8)*	c.1186A>G	p.(T396A)	8	Benign	Neutral	Tolerated	0
*KCNA5 (Exon 1)*	c.125T>A	p.(L42H)	1	Benign	Neutral	Tolerated	0
*KCNA5 (Exon 1)*	c.253C>A	p.(L85M)	1	Benign	Neutral	Tolerated	0
*KCNA5 (Exon 1)*	c.340A>C	p.(T114P)	1	Benign	Neutral	Tolerated	0
*KCNA5 (Exon 1)*	c.385C>G	p.(L119V)	2	Benign	Neutral	Damaging	1
*KCNA5 (Exon 1)*	c.509C>G	p.(P170R)	1	Probably Damaging	Pathological	Damaging	3
*KCNA5 (Exon 1)*	c.551G>C	p.(R184P)	1	Probably Damaging	Pathological	Damaging	3
*KCNA5 (Exon 1)*	c.1733G>A	p.(R577K)	1	Benign	Neutral	Tolerated	0

These results are considered damaging if the score is equal or greater than two.

**Table 3:** Results from four different bioinformatic programs used to predict the effect on the splicing process in *BMPR2* gene (NNSplice, NetGene2, Splice View and HSF Human).

*Sequence variants*	*NNSplice*	*NetGene2*	*Splice View*	*HSF Human*	*Score*
**c.1-301G>A**	The WT consensussequenceis not recognized	Score for the aceptor siteincreases from 25 to 26	The WT consensus sequenceis not recognized	Neutral	1
**c.190A>C (p.(S64G))**	Neutral	The WT consensus sequenceis not recognized	A new donor siteis created	Score for donor andacceptor site decreases	2
**c.229A>T (p.(I77L))**	The WT consensussequenceis not recognized	Score for the main donor siteincreases from 31 to 34	Neutral	A new acceptor siteis created	2
**c.251G>T (p.(C84F))**	Score for the aceptor siteincreases from 87 to 89	Score for the main acceptor sitedecreases from 33 to 27	Neutral	The main donor siteis not recognized	3
**c.259C>T (p.(H87Y))**	Score for the aceptor sitedecreases from 87 to 86	Score for the main acceptor sitedecreases from 33 to 30	Neutral	The main donor site is notrecognized and theacceptor increase	3
**c.275A>T (p.(Q92L))**	Neutral	Score for the main acceptor sitedecreases from 33 to 25	Neutral	Score for donor and acceptorsite increases	2
**c.327G>C (p.(Q109Q))**	Neutral	Score for the main donor sitedecreases from 79 to 76	Neutral	The main donor site isnot recognized	2
**c.419-43delT**	Neutral	Neutral	Neutral	Neutral	0
**c.484G>C (p.(A162P))**	Score for the aceptor sitedecreases from 80 to 66	Neutral	Neutral	The main donor site is notrecognized	2
**c.529+37C>G**	A new acceptor siteis created	Neutral	Neutral	A new acceptor site is created	2
**c.529+53A>G**	Neutral	Neutral	Neutral	A new acceptor site is created	1
**c.529+139A>T**	Neutral	Neutral	Neutral	Score for donor site decreasesand the acceptor site increase	1
**c.530-24G>T**	Neutral	Score for the main donor siteincreases from 83 to 86 and themain acceptor site increasesfrom 77 to 82	Neutral	The donor and acceptor sitesis not recognized	2
**c.622+103C>G**	Neutral	Neutral	Neutral	The main donor site is not recognized	1
**c.633A>G (p.(R211R))**	Neutral	Score for the main donor siteincreases from 92 to 94	Neutral	The main donor site is notrecognized and the acceptor decrease	2
**c.637C>A (p.(R213R))**	Neutral	Score for the main acceptor sitedecreases from 20 to 18	Neutral	Score for donor site increasesand a new acceptor site is created	2
c.654T>A (p.(Y218*))	Neutral	Score for the main donor siteincreases from 92 to 94 andthe main acceptor sitedecreases from 20 to 18	Neutral	Score for the main acceptorsite decrease	2
**c.790G>A (p.(D264N))**	Neutral	Score for the main donor sitedecreases from 94 to 92	Neutral	The main donor site is not recognized	2
**c.835G>T (p.(V278V))**	Neutral	Neutral	Neutral	Score for donor site decreasesand the acceptor site increase	1
**c.853-22T>C**	Neutral	Score for the main donor sitedecreases from 87 to 86	Neutral	Neutral	1
**c.968+117G>A** *BMPR2*	Neutral	Neutral	Neutral	The donor and acceptor sitesis not recognized	1
**c.968-124_968-122delTCT**	Neutral	Neutral	Neutral	Neutral	0
c.893G>A (p.(W298*))	Neutral	Score for the main donor andacceptor site decreases	The WT consensus sequenceis not recognized	The main donor site increase anda new acceptor site in created	2
**c.981T>C (p.(P327P))**	The WT consensussequenceis not recognized	Score for the main donor sitedecreases from 100 to 99	Neutral	A new donor site is created	2
**c.1021G>A (p.(V341M))**	Neutral	Neutral	The WT consensus sequence isnot recognized	The main donor site is not recognized	2
**c.1414-84C>T**	Neutral	Neutral	Neutral	Score for the main acceptor site increases	1
**c.1467G>A (p.(E489E))**	Neutral	Score for the main donor siteincreases from 89 to 93	Neutral	A new acceptor site is created	2
**c.2034G>A (p.(K678K))**	Neutral	Neutral	Neutral	The main donor site is not recognized	1
**c.2324G>A (p.(S775N))**	Neutral	Neutral	Neutral	The main donor site is not recognized	1
**c.2811G>A (p.(R937R))**	Neutral	Neutral	Neutral	The main donor site is not recognized	1

These results are considered positive if the score is equal or greater than two.

**Table 4:** Results from four different bioinformatic programs used to predict the effect on the splicing process in *ACVRL1* gene (NNSplice, NetGene2, Splice View and HSF Human).

*Sequence variants*	*NNSplice*	*NetGene2*	*Splice View*	*HSF Human*	*Score*
**c.24A>T (p.(K8N))**	Score for the mainacceptor site increasesfrom 66 to 76	Neutral	Neutral	The main donor and acceptor sitesare not recognized	2
**c.176A>T (p.(E59V))**	Neutral	Neutral	Neutral	The main donor site is not recognizedand a new acceptor site is created	0
**c.313+11C>T**	Neutral	Neutral	Neutral	Score for acceptor site decreases	1
**c.313+20C>A**	Neutral	Neutral	Neutral	Score for acceptor site increase	1
**c.476A>T (p.(E159V))**	The WT consensus sequenceis not recognized	The WT consensus sequenceis not recognized	Neutral	The main donor site is not recognizedand acceptor site increase	0
**c.478delT** (p.(S160Pfs*5))	Neutral	Neutral	Neutral	The main donor site is not recognized	1
**c.673A>T (p.(S225C))**	Score for the main donor siteincreases from 95 to 99	Score for the main donor siteincreases from 39 to 55	The WT consensus sequenceis not recognized	The main donor site is not recognizedand a new acceptor site is created	2
**c.1047+31C>A** *ACVRL1*	The WT consensus sequenceis not recognized	The WT consensus sequenceis not recognized	Neutral	The main donor site is not recognized	1
**c.1186A>G (p.(T396A))**	The WT consensus sequenceis not recognized	Neutral	Neutral	Score for the main donor site increases	0
**c.1246+41G>C**	The WT consensus sequenceis not recognized	Neutral	Neutral	The main acceptor site is not recognized	0
**c.1502+7A>G**	Neutral	Score for the main donor sitedecreases from 83 to 70	The WT consensus sequenceis not recognized	Neutral	1

These results are considered positive if the score is equal or greater than two.

**Table 5:** Results from four different bioinformatic programs used to predict the effect on the splicing process in *KCNA5* gene (NNSplice, NetGene2, Splice View and HSF Human).

*Sequence variants*	*NNSplice*	*NetGene2*	*Splice View*	*HSF Human*	*Score*
**c.125T>A (p.(L42H))**	The WT consensus sequenceis not recognized	The WT consensus sequenceis not recognized	The WT consensus sequenceis not recognized	The WT consensus sequenceis not recognized	0
**c.253C>A (p.(L85M))**	The WT consensus sequenceis not recognized	The WT consensus sequenceis not recognized	The WT consensus sequenceis not recognized	The WT consensus sequenceis not recognized	0
**c.340A>C (p.(T114P))**	The WT consensus sequenceis not recognized	The WT consensus sequenceis not recognized	The WT consensus sequenceis not recognized	The WT consensus sequenceis not recognized	0
**c.385C>G (p.(L119V))**	The WT consensus sequenceis not recognized	The WT consensus sequenceis not recognized	The WT consensus sequenceis not recognized	Neutral	0
**c.477G>C (p.(L159L))**	The WT consensus sequenceis not recognized	The WT consensus sequenceis not recognized	The WT consensus sequenceis not recognized	Score for the main acceptorsite increase	1
**c.509C>G (p.(P170R))**	The WT consensus sequenceis not recognized	The WT consensus sequenceis not recognized	The WT consensus sequenceis not recognized	The WT consensus sequenceis not recognized	0
**c.551G>C (p.(R184P))**	The WT consensus sequenceis not recognized	The WT consensus sequenceis not recognized	The WT consensus sequenceis not recognized	The WT consensus sequenceis not recognized	0
c.622G>T (p.(E208*))	The WT consensus sequenceis not recognized	The WT consensus sequenceis not recognized	The WT consensus sequenceis not recognized	The main donor site is notrecognized and acceptor site increase	1
**c.1733G>A (p.(R577K))**	The WT consensus sequenceis not recognized	The WT consensus sequenceis not recognized	The WT consensus sequenceis not recognized	The WT consensus sequenceis not recognized	0
**c.1842+22T>G**	The WT consensus sequenceis not recognized	The WT consensus sequenceis not recognized	The WT consensus sequenceis not recognized	The WT consensus sequenceis not recognized	0
**c.1842+52A>T**	The WT consensus sequenceis not recognized	The WT consensus sequenceis not recognized	The WT consensus sequenceis not recognized	The WT consensus sequenceis not recognized	0
**c.1842+508A>T**	The WT consensus sequenceis not recognized	The WT consensus sequenceis not recognized	The WT consensus sequenceis not recognized	The WT consensus sequenceis not recognized	0

These results are considered positive if the score is equal or greater than two.

**Figure 2:**
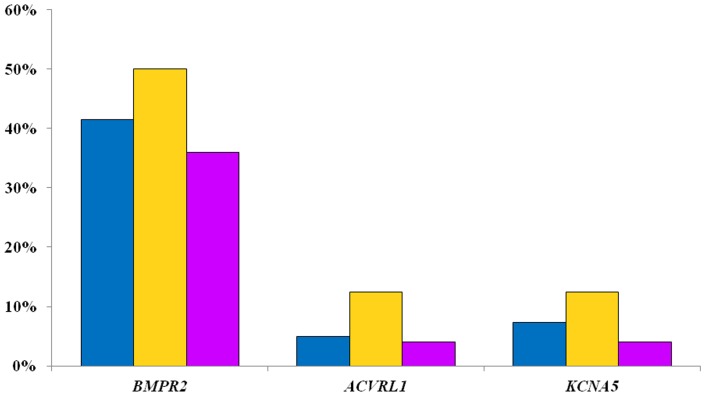
Frequency of pathological mutations found in our patients (blue all patients, yellow IPAH, purple APAH). *BMPR2* showed the greatest number of mutations.

After the combination of all software, we found 22 pathological mutations in 21 patients, with a frequency of 51%. Most of the mutations were found in *BMPR2* gene (41.5%), followed by *KCNA5* (7.3%) and, finally, *ACVRL1* (4.9%). These results are shown in Figure 2. IPAH patients were carriers of *BMPR2* mutations in 8 cases (50%), two cases for *KCNA5* gene (12.5%) and 2 cases for *ACVRL1* gene (12.5%) (Figure 2). The 62.5% of patients (10patients) with IPAH had at least one pathogenic mutation in some of these genes. On the other hand, 44% (11 patients) of APAH patients showed pathogenic mutations (36% in *BMPR2* gene, 4% in *ACVRL1* gene and 4% in *KCNA5* gene).

In our cohort of patients we found 5 patients with more than one pathogenic mutation in *BMPR2* or in combination with *KCNA5* or *ACVRL1* genes (Table 6). All of these mutations are described for first time in this study, except c.529+37C>G.

**Table 6:** List of patients with several pathogenic mutations in the studied genes.

Patient	*BMPR2*	*ACVRL1*	*KCNA5*	*PAH Type*
**14.09**	c.251G>T (p.(C84F)), c.259C>T (p.(H87Y))^a^, c.981T>C (p.(P327P)) a	–	–	Associated
**14.17**	c.530+37C>G a	–	c.551G>C (p.(R184P))	Idiopathic
**17.01**	c.893G>A (p.(W298*))	c.24A>T (p.(K8N)) a	–	Idiopathic
**PO.15**	c.229A>T (p.(I77L))^a^, c.633A>G *(*p.(R211R)) a	–	–	Idiopathic
**PO.16**	c.327G>C *(*p.(Q109Q)) a, c.1021G>A (p.(V341M))	–	–	Associated

a These mutationsare considered pathogenic because they could produce alterations in the splicing process, according to *in silico* analysis.

### Association with clinical features and hemodynamic parameters

We analyzed clinical features and hemodynamic parameters comparing the group of patients harboring a pathogenic mutation with those patients with no mutation. The parameters included were: gender, age at diagnosis, mPaP (mean pulmonary pressure), sPaP (systolic pulmonary pressure), PVR (pulmonary vascular resistence), CI (cardiac index), 6MWT (6 minute walking test) and type of PAH (IPAH vs APAH). Variables were categorized according to the best cut off point by ROC curve.

The association of genotype with clinical and hemodynamics parameters showed statistically significant differences in mPaP (p = 0.043) and PVR (p = 0.043). Patients carrying a mutation had a higher value for these two parameters than non-carriers. In opposition, patients carrying a mutation had a significantly lower CI (p = 0.04) and 6MWT (p = 0.02). These results are shown in table 1. We did not found significant differences between gender for the presence of mutations (p = 0.216), mean age of onset of first symptoms (p = 0.437) and sPaP (p = 0.448). Pathological mutations were seen in 10 patients with IPAH and 10 patients with associated PAH without statistical differences (p = 0,222). Clinical and hemodynamic parameters did not show any significant difference between associated and idiopathic PAH.

The mean follow-up period was 14 months. Three patients died during this time (2 APAH, 1 IPAH), so it was not possible to compare groups. Two of the three deceased patients present two pathogenic mutations in *BMPR2* gene. The other patient was carrier of a pathogenic mutation, (p.(A162P)) in *BMPR2* gene and showed several polymorphisms in *ACVRL1* (c.1047+31C>A, c.1186A>G (p.(T396A)), c.1502+7A>G) and *KCNA5* (c.1842+508A>T) genes.

## Discussion

This study was designed to establish the prevalence of mutations found in certain genes potentially involved in the pathogenesis of PAH. We detected 53 different nucleotide changes in *BMPR2, ACVRL1* and *KCNA5* genes in 40 out of 41 PAH patients. Twenty two of these mutations (found in 51% of patients) were considered pathogenic according to the *in silico* analysis that was performed with several programs to reach a high reliability [21].

The mutational frequency for *BMPR2* gene in sporadic PAH range from 10–20%, as referred in previous studies [22], [23], [24].However, we found that 50% of our IPAH patients had a pathogenic mutation in *BMPR2* gene,higher than expected and corresponding to the highest value described until now. The percentage of mutations found in APAH was 36%, which is lower but also significantly elevated. Consider the suggestion made by Pfarr *et al* [25] that as soon a genetic defect had been identified in PAH patients they must be classified as familial PAH, it could be interesting to perform segregation analysis in order to confirm the familial nature of the disease in these families.

We identified two hot spots for mutations in exon 3 and exon 6 for *BMPR2* gene. These exons are located in a very important area rich in cysteine residues and therefore any mutation here could affect the catalytic ability of the BMPs, the ligand of *BMPR2*, disrupting the Smad signaling pathway [2], [23], [26], [27], [28]. These mutations may introduce subtle changes in the structure of the protein and might interfere with the downstream signaling of the BMP pathway [29]. It has been hypothesized that an imbalance of increased TGF-β levels and decreased BMP signals induced by *BMPR2* mutations leads to PAH [30]. Exon 6 is located in the N-terminus of a serine-threonine kinase domain which is formed by conserved subdomains that includes exons 6 to 11 of the gene. This region, responsible for binding adenosine triphosphate (ATP), is characterized by distinctive patterns of conserved residues. Mutations located here could produce heterogeneous defects for signaling activity by binding preventing of ATP and altering the functionality of the protein. We also detected several mutations in the C-terminus region for this serine-threonine kinase domain, which is involved in substrate recognition and initiation of the phosphorylation relay. In addition, one missense mutation and one synonymous change, which seems to alter the splicing process according to *in silico* analysis, were identified in exon 8.Finally, we found the synonymous mutation p.(E489E) in exon 11, which is predicted to produce alterations at splicing level. An invariant arginine at position 491 in the protein is located around this point, which is essential for signaling [31], thereby making this area of special interest.

Synonymous mutations have always been considered safe, but they could cause serious physiological effects as they can interfere in the splicing accuracy, translation fidelity, mRNA structure and protein folding. Even, these mutations may decrease the half-life of mRNA, leading to a downregulation of the protein expression [32]. Different synonymous mutations, both new and already described, were seen in 46% of our patients and the 62.5% of these synonymous mutations are considered pathogenic and were not found in controls. It could be interesting to analyze synonymous mutations and intronic deletions [23] with a functional approach since no studies have been performed for these changes.

Regarding to the *ACVRL1* gene, several mutations have usually been described associated with HHT. Girerd *et al* describe a mutational frequency for this gene of 2.3% in IPAH patients from the French PAH Network, lower than 12.5% found in our patients [33]. Probably the small size of our series may explain these differences. When we compared the data for IPAH in this paper against our results, we found a higher mutational rate for *BMPR2* and *ACVRL1* genes (59% vs 16.6%), finding only one mutation (p.(S160P)fs*5) in *ACVRL1* in one APAH patient. Selva-O’Callaghan *et al*, who studied mutational load for *ACVRL1* gene in APAH to connective tissue disease patients [34], did not found any mutation. These findings suggest that *ACVRL1* gene do not have a significant role in APAH patients.

The p.(S225C) mutation is located in exon 6 of *ACVRL1* gene and it is placed in the serine-threonine kinase domain. Mutations in this region, a conserved serine–threonine kinase domain, have been associated with a higher risk of PAH in childhood and could affect the downstream SMAD signaling pathway as *BMPR2* [35], [36]. The novel missense mutation, p.(T396A) was detected in 8 patients and does not appear in the control group. Functional studies from Abdalla et al [36] demonstrated that mutations located in this highly conserved protein domain may cause protein misfolding [36], [37] and intracellular degradation, explaining the lack of surface expression of mutant proteins [36]. Although it has been classified as nonpathogenic, it would be interesting to determine its functionality.

Few mutations have been described in highly conserved aminoacid residues of the *KCNA5* gene in PAH patients [38]. Several *KCNA5* gene mutations have been involved in atrial fibrillation, a common cardiac arrhythmia, in 1.95% of patients with absence of known predisposing factors [39]. We detect pathological mutations in this gene in 7.3% of total patients. For IPAH patients this value raises to 12.5%, but only one mutation was identified in one APAH patient.

The p.(P170R), p.(R184P) and p.(E208*) mutations in *KCNA5* gene are located within the T1 domain, which is highly conserved. The T1 domain is largely responsible for tetramerization and governs channel interaction by cytoplasmic regulatory subunits KVα and KVβ. Mutations located in this region have been shown to disrupt both KVα-KVβ with deleterious consequences on channel gating, protein expression [40] or cause both hyperpolarizing and depolarizing shifts on the activation relationship [41], [42]. T1 domain has also been associated with other aspects of channel function as the interaction with S1 domain or the influence in gating properties and voltage sensitivity of KV channels. Furthermore, the p.(E208*) mutation is close to S1 domain and potentially can disrupt the creating side portals between the T1 domain and the pore. Even mutations that have been identify as no harmful for the protein and did not appear in controls, as p.(L42H), p.(T114P) and p.(R577K) could introduce subtle changes in the structure of the protein and might interfere with the proper operation of the Kv1.5 channels, since they are located between the 5′UTR region of the gene and heteromerization domain, where the association between different proteins occurs [38].

We found statistical differences for mPaP, PVR, CI and 6MWT when compared hemodynamics and clinical parameters between patients with and without pathogenic mutations. Patients who harbor mutations show higher values for mPaP and PVR. Conversely, values for CI and 6MWT were significantly smaller. These results seem to show that patients with mutations have a more severe disease and perhaps worse prognosis. Otherwise,Pfarr *et al* found significant differences only for a low PVR value [25]. On the other hand, no differences in these parameters were seen according to PAH type. Liu D *et al* have described that gender influences the phenotype in PAH patients with *BMPR2* mutations, being more severe in males, but we did not confirm this fact in our results [43]. Previous studies indicate that PAH patients carrying a mutation have an onset approximately 10 years earlier than non-carriers [31], but our results did not confirm this finding. The vast majority of these variations are private, so it makes very difficult to establish a correlation between the phenotype and one particular mutation. For this reason, genotype-phenotype correlation is made according to all mutations found in a group of patients.

We described for the first time 5 patients with multiple mutations, three of them with two or more mutations in *BMPR2* gene. Two patients, both with IPAH, were carriers of mutations in two genes, *BMPR2* and *KCNA5* genes in one case and *BMPR2* and *ACVRL1* genes in the other one. This genetic heterogeneity reinforces the complex pathogenicity of this disease, with several ways of actuation. The molecular mechanisms of PAH are not clearly understood but multiple factors are involved in the development of this disease and several genes could be mutated, so it is not surprising that one patient may require mutations in several genes to develop PAH. The small number of patients with various mutations does not allow comparisons, but 3 of these 5 patients had a younger age at diagnosis and 2 of them died during follow-up which may suggest a worse prognosis.

Obviously, the main limitation of our study is the small number of patients, although the low incidence of PAH and some cases that did not consent the inclusion in this study, did not allow us to have a larger series. The comprehensive study carried out and complete follow-up of all cases add value to our results.

In summary, we present a series of patients with idiopathic and associated PAH with a high percentage of mutations in *BMPR2* and lower in *ACVRL1* and *KCNA5* genes, some of them not previously described, showing some clinical and hemodynamic differences which suggest that the presence of these mutations may be associated with more severe disease. There is no doubt that other genes are involved in the pathogenesis of PAH and will be important to know the role they play in the development of this disease. Perhaps the presence of more than one mutation increases the risk of develop it. As in other pathologies with genetic basis, PAH may be caused by a total mutational load of the genes involved in. This genetic heterogeneity, when known, may allow us to establish a correlation with the severity and course of the disease. The more we known about the pathways involved the best we can design the treatment.
